# Controlled Tensile Behavior of Pre-Cured PDMS via Advanced Bonding Techniques

**DOI:** 10.3390/polym17010103

**Published:** 2025-01-02

**Authors:** Minwook Park, Jungho Shin, Seunghyun Lee

**Affiliations:** 1 Department of Mechanical System Design Engineering, Seoul National University of Science and Technology, Seoul 01811, Republic of Korea; qkralshrl98@seoultech.ac.kr; 2 Department of Mechanical Engineering, Texas A&M University, College Station, TX 77843, USA; jungho.shin0912@tamu.edu

**Keywords:** polydimethylsiloxane (PDMS), mechanical testing, crosslink density, PDMS-PDMS bonding technique, tunable tensile properties

## Abstract

Polydimethylsiloxane (PDMS) is extensively employed in applications ranging from flexible electronics to microfluidics due to its elasticity, transparency, and biocompatibility. However, enhancing interfacial adhesion and tensile properties remains a challenge for applications demanding high mechanical stability. To this end, this study introduced a novel bonding technique using crosslinkers as adhesive layers to improve the mechanical performance of PDMS. By adjusting the crosslink density at the PDMS-PDMS interfaces, we achieved substantial improvements in tensile properties and interfacial adhesion. Our findings revealed that, under specific conditions, a particular mixing ratio significantly enhances the elastic modulus and interfacial stability. Notably, the elastic modulus of PDMS with a tailored crosslink density increased by approximately 760% compared to that achieved with a simple bonding method. This study demonstrated an effective strategy for tailoring the interfacial properties of PDMS by adjusting the crosslink density, offering a pathway to enhance material design for applications requiring advanced mechanical performance and stability.

## 1. Introduction

Polydimethylsiloxane (PDMS) is a widely recognized silicone elastomer with exceptional elasticity, transparency, biocompatibility, thermal stability, and tunable material properties [1,2,3,4]. These unique characteristics and versatility make PDMS an essential material in fields ranging from wearable devices [5] to biomedical engineering [6,7,8] and microfluidics [9]. Reliable PDMS bonding is crucial for applications requiring structural integrity and functionality over repeated use. PDMS bonding methods are generally categorized into physical bonding (e.g., pressure bonding and thermal bonding) and chemical bonding (e.g., oxygen plasma treatment and UV/ozone exposure), each providing varying degrees of strength and durability depending on the specific application. Notably, the effectiveness and reliability of systems such as stretchable devices heavily rely on the strength and durability of PDMS bonding. Park et al. demonstrated that pressure bonding enhances PDMS bonding, thereby extending the performance of stretchable electrodes to withstand 1000-cycle prolonged stress conditions [10]. Since strong and durable PDMS-PDMS interfaces are essential for achieving mechanical reliability and supporting complex geometric designs under harsh conditions, extensive research has been conducted to explore different physical and chemical bonding techniques between PDMS layers [11,12,13].

Physical bonding techniques, such as pressure and thermal bonding, enhance the mechanical stability of PDMS structures by improving the physical contact and interactions between layers. Chemical bonding techniques, such as plasma treatment, UV/ozone treatment, and corona discharge, modify the chemical structure of PDMS surfaces by introducing reactive species or adding polar functional groups [14,15]. Although these conventional bonding approaches can enhance mechanical properties, they lack the flexibility to selectively tune these properties. For PDMS, which is used in a wide range of applications, the selective control of its mechanical properties could enable more efficient and application-specific designs.

To this end, we investigated a method for selectively enhancing the tensile properties of PDMS systems by engineering the interface between PDMS layers. Specifically, we developed a mechanism to independently tune the mechanical properties of the bulk and interfacial regions by adjusting the crosslink density within the interface between PDMS layers. Crosslink density plays a critical role in determining mechanical properties, allowing control over a material’s strength, flexibility, and durability [2]. By regulating the crosslink density at the interface, we demonstrated the ability to selectively tailor the tensile properties of the overall PDMS system.

We herein explored the potential of using a crosslinker as an adhesive layer in PDMS-PDMS bonding to control the tensile properties of pre-cured PDMS. We hypothesized that adjusting the crosslink density at the interface would allow for tailored mechanical characteristics of the bonded PDMS, particularly in thin layers. Our approach involved depositing a crosslinker on the interface of pre-cured PDMS layers with low crosslink density, where the polymer chains retained sufficient mobility, followed by post-curing. This strategy maximized chain interdiffusion and crosslink formation at the interface, thereby enhancing the tensile properties of the bonded PDMS by forming a robust crosslinked network specifically at the interface.

To assess the effectiveness of this method, swelling experiments were performed to verify whether the crosslinker successfully increased the crosslink density at the interface. The degree of crosslinking was quantified using the Flory–Rehner equation, providing quantitative insights into the crosslinking process. Subsequently, tensile tests were conducted to examine the effect of variations in the crosslink density at the interface on the overall tensile behavior and mechanical properties of PDMS. The elastic modulus of PDMS was quantitatively measured, offering essential baseline properties useful in applied research fields.

This study provides a versatile framework for advancing the use of PDMS in fields requiring tailored mechanical behaviors, such as flexible electronics and microfluidic devices, where structural integrity and performance are of paramount importance.

## 2. Materials and Methods

### 2.1. Fabrication of PDMS Specimens

The PDMS (polydimethylsiloxane) specimens were prepared using Sylgard 184 (Dow Corning, Midland, MI, USA). To evaluate the effect of crosslinker density on adhesion, the base and crosslinker were mixed at weight ratios of 10, 20, 30, and 40 (base/crosslinker by weight). Each mixture was thoroughly stirred for 5 min to ensure homogeneity and then cast into molds to form the test specimens. The specimens were degassed in a vacuum chamber for 60 min to remove trapped air bubbles and cured in an oven at 70 °C for 1 h.

**Figure 1:**
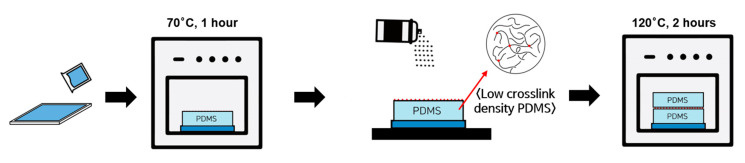
Preparation process for PDMS_AL_ specimens. PDMS specimens were bonded with the crosslinker as an adhesive layer, deposited uniformly via air gun spraying. The assembled layers underwent an additional curing step at 120 °C for 2 h to promote interfacial crosslinking.

The crosslinker was applied as an adhesive layer using an air gun to ensure uniform deposition. It was sprayed at a flow rate of 0.026 g/s from a distance of 30 cm for 30 s. After crosslinker deposition, the PDMS layers were bonded by overlaying a pretreated PDMS layer onto another cured PDMS layer. The assembled samples were then subjected to additional curing at 120 °C for 1 h to facilitate crosslinking at the interface (Figure 1). For comparison, three bonding techniques were utilized: (1) simply bonded PDMS specimens without post-treatment (referred to as “PDMS_SB_”), (2) post-cured specimens without crosslinker deposition (referred to as “PDMS_PC_”), and (3) post-cured specimens treated with a crosslinker as an adhesive layer (referred to as “PDMS_AL_”).

### 2.2. Swelling Ratio Measurements

Swelling experiments were conducted to assess the crosslink density of PDMS specimens fabricated with the specified base/crosslinker ratios (i.e., 10, 20, 30, and 40, hereafter referred to as the “mixing ratio”) and bonded using three different techniques. Dry specimens (30 mm × 30 mm × 2 mm) were immersed in silicone oil (KF-96-10cs, Shin-Etsu Chemical Co., Ltd. Tokyo, Japan) to observe their swelling behavior. The swelling ratio was calculated by weighing the swollen specimens at predetermined time intervals over 7 days (168 h). The weight swelling ratio *S* was determined using the following equation:

$$S\left(\%\right)=\frac{\left(W_{s}-W_{d}\right)}{W_{d}}\times 100\%$$

where *W_s_* denotes the weight of the swollen specimen, and *W_d_* denotes the weight of the dry specimen. This measurement provides the extent of solvent uptake, which is correlated to the crosslinking density of the polymer.

### 2.3. Crosslink Density Estimation

The crosslink density was estimated using the method described by Sortiri et al. [7]. Specifically, the weights of the specimens from the swelling experiment were measured once equilibrium was reached (~160 h). The crosslink density was calculated using the Flory–Rehner equation:

$$\nu =\frac{-\left(\ln\left(1-\Phi_{p}\right)+\Phi_{P}+\chi \Phi_{P}^{2}\right)}{V\left(\Phi_{P}^{\frac{1}{3}}-\frac{\Phi_{P}}{2}\right)}$$

where *Φ_P_* is the volume fraction of the swollen polymer, *V* is the molar volume of the solvent (silicone oil), and χ is the Flory–Huggins polymer–solvent interaction parameter, assumed to be zero (χ *≈ 0*) herein due to the similar chemical compositions of silicone oil and PDMS. The molar volume of the silicone oil, 797.9 cm^3^/mol, was calculated using its molecular weight of 770 g/mol and density of 0.935 g/cm^3^. The volume fraction of the swollen polymer was determined using the average density of PDMS, 1.03 g/cm^3^, and the density of the silicone oil.

### 2.4. Tensile Testing

Tensile tests were performed on a universal tensile machine (MINOS-020, MTDI Inc., Daejeon, Republic of Korea) with a 2 kN load cell (DSCH, Bongshin Loadcell Co., Ltd., Osan, Republic of Korea). The specimens measured 60 mm × 30 mm × 1 mm (length × width × thickness). A constant strain rate of 100%/min was applied throughout the test, and the strain (ε) along the tensile direction was calculated as ε = δ/L, where δ represents the cross-head displacement, and L is the initial length of the specimen. Engineering stress was calculated by dividing the applied force by the initial cross-sectional area of the specimen, and the elastic modulus was estimated from the slope of the stress–strain curve up to a strain of 30%. All mechanical tests were conducted at room temperature.

## 3. Results and Discussion

### 3.1. Modulation of PDMS Tensile Properties by Crosslink Density at Interface

**Figure 2:**
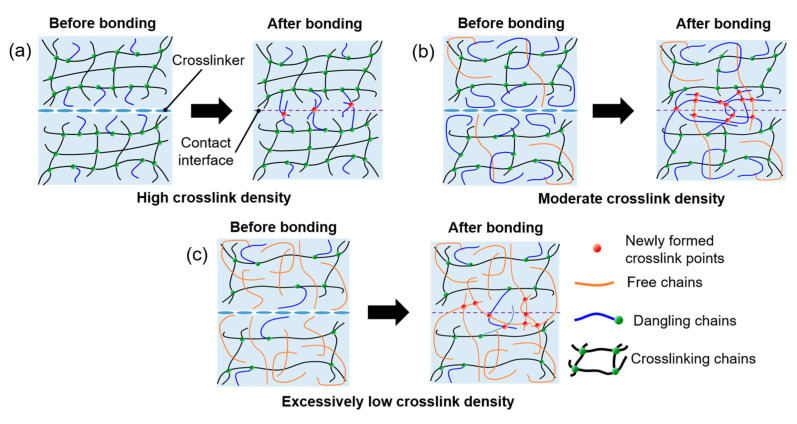
Schematic of PDMS layers, illustrating impact of varying crosslink densities on interfacial adhesion. (**a**) High crosslink density restricts chain length and mobility, hindering chain interdiffusion and covalent bond formation across interface. (**b**) Moderate crosslink density allows for effective interdiffusion and covalent bonding, leading to strong interfacial adhesion with enhanced mechanical integrity. (**c**) Low crosslink density increases interpenetration, but bonding is primarily limited to free chains at interface, reducing stress transfer efficiency. Controlling bulk crosslink density is critical for achieving strong interfacial bonding and improved mechanical properties.

The tensile properties of the entire system were significantly influenced by the crosslink density of PDMS at the interface (bonding layer). Figure 2 shows the microstructures of the PDMS bonding layers with various crosslink densities. In bulk PDMS, fewer crosslinking points are formed as the crosslink density decreases, thereby increasing the distance between them and limiting the development of a complete 3D network. With fewer connections, the polymer chains experience reduced constraints, leading to more free chains and longer dangling ends, which enhance chain mobility. This increased mobility allows polymer chains from adjacent PDMS layers to penetrate and entangle more extensively (i.e., chain interdiffusion), thereby strengthening the interfacial interactions [16].

Introducing a crosslinker at the interface promotes the formation of additional covalent bonds and significantly increases the crosslink density at the interface (Figure 2b). This observation aligns with previous studies, which show that the structure of crosslinking agents significantly influences the formation of crosslinked networks and the mechanical properties of PDMS [17]. These additional covalent bonds enhance the chemical connectivity between the layers, reducing the likelihood of interfacial failure under tensile stress. Furthermore, the denser interfacial network minimizes structural defects such as voids and microcracks, ensuring a more uniform stress distribution during mechanical deformation. As previously demonstrated, enhanced chain interdiffusion at the interface further promotes entanglement and covalent bonding, thereby improving both the interfacial adhesion and tensile properties. The interplay between chain interdiffusion and covalent bonding establishes a robust interfacial network, contributing to improved bonding between the PDMS layers and overall tensile properties.

However, when the crosslink density is excessively low, as shown in Figure 2c, the proportion of free chains capable of interdiffusion increases, whereas the number of dangling chains decreases. Although this led to higher chain mobility and extensive interpenetration of the polymer chains, the tensile properties did not improve. With a relatively low crosslink density, crosslinking remained confined to localized areas around the interface, disconnected from the 3D network of bulk PDMS. Consequently, stress transfer between polymer networks during tensile loading was limited, restricting the overall mechanical performance [18,19]. These limitations highlight the importance of controlling the crosslink density of bulk PDMS to achieve an appropriate balance that improves interfacial bonding, facilitates stress transfer, and enhances tensile strength.

### 3.2. Swelling Ratio Measurement and Crosslink Density Estimation

Swelling experiments were conducted to determine whether the initial crosslink density of the bulk PDMS used in adhesion affects the interfacial crosslinking when a crosslinker is applied as an adhesive. Swelling experiments are commonly used to assess the crosslink density of elastomers, where an increased crosslink density results in a lower swelling ratio due to reduced free volume, limited chain mobility, and higher energy requirements for swelling, which restrict solvent diffusion [7].

**Figure 3:**
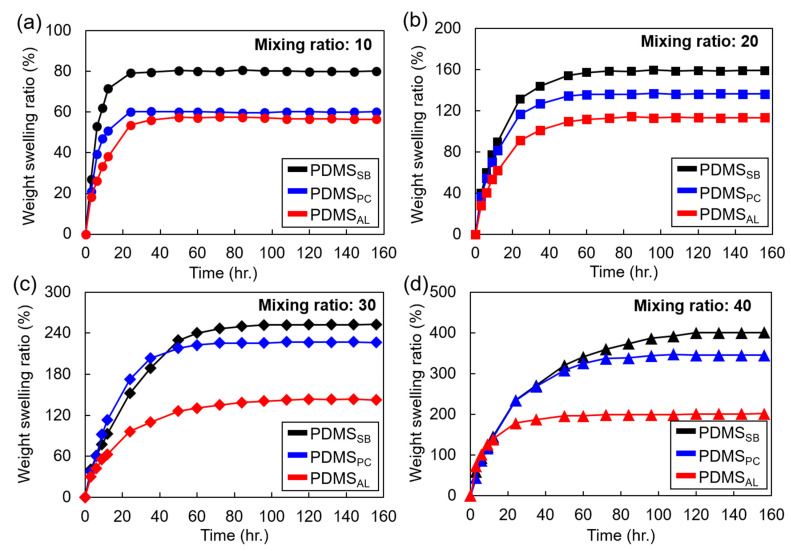
Representative weight swelling ratio as function of time with different mixing ratios of bulk PDMS used for adhesion and various bonding techniques: (**a**) mixing ratio of 10, (**b**) mixing ratio of 20, (**c**) mixing ratio of 30, and (**d**) mixing ratio of 40. Difference in scales among four plots should be noted.

Figure 3 illustrates the weight swelling ratios of the PDMS samples prepared using the three different bonding methods over time at various mixing ratios. Here, PDMS_AL_, PDMS_PC_, and PDMS_SB_ represent the samples in which a crosslinker was applied at the interface, post-cured specimens without crosslinker deposition, and simply bonded specimens, respectively. The swelling ratios of PDMS_AL_ (red lines) across all mixing ratios were consistently lower than those of PDMS_SB_ (black lines). The decrease in the swelling ratio became more pronounced as the PDMS mixing ratio (base/crosslinker) increased, indicating a lower crosslinker concentration in the PDMS. Given that PDMS_AL_, PDMS_PC_, and PDMS_SB_ reflect different bonding methods and therefore different crosslink densities, these results suggest that the crosslink density of the PDMS bonding layer is crucial for interfacial bonding when a crosslinker is used as an adhesive layer.

**Figure 4:**
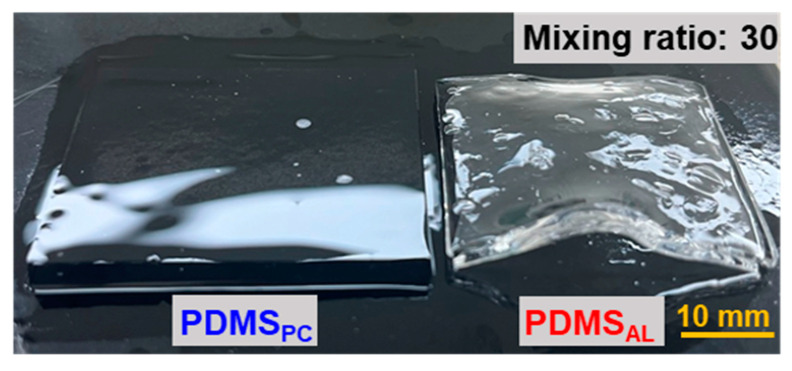
Photos of swelling deformation at equilibrium. The PDMS_PC_ specimen, post-cured without a crosslinker adhesive layer, exhibits a homogeneous microstructure, resulting in uniform swelling deformation (**left**). In contrast, the PDMS_AL_ specimen, treated with a crosslinker adhesive layer, displays an inhomogeneous structure due to increased interfacial crosslink density, leading to uneven swelling and warping (**right**).

By comparing PDMS_AL_ and PDMS_PC_ (blue lines in Figure 3), the reduced swelling ratio observed in PDMS_AL_ can be attributed to changes in crosslink density specifically at the interface rather than within the bulk PDMS involved in bonding. For PDMS_PC_, uncured chains from the relatively short curing time likely underwent additional crosslinking within the bulk material during post-curing, resulting in a slight increase in the bulk crosslink density. Despite this increase in the crosslink density of PDMS_PC_, the reduction in its swelling ratio remained nearly negligible compared to that of PDMS_AL_, suggesting that the reduced swelling ratio in PDMS_AL_ was primarily due to structural modifications at the interface rather than changes in the bulk PDMS microstructure. These findings are supported by the warping observed in the PDMS_AL_ samples during swelling (Figure 4), which resulted from changes in the interfacial crosslink density that caused visible deformation. Warping, which is a bending or twisting deformation caused by internal stresses, typically occurs in samples with altered interfacial crosslink densities. As shown in Figure 4, the warping suggests significant structural modification at the interface and highlights the differences between the bulk and interfacial regions. Table 1 summarizes the equilibrium swelling ratios of the test specimens.

**Figure 5:**
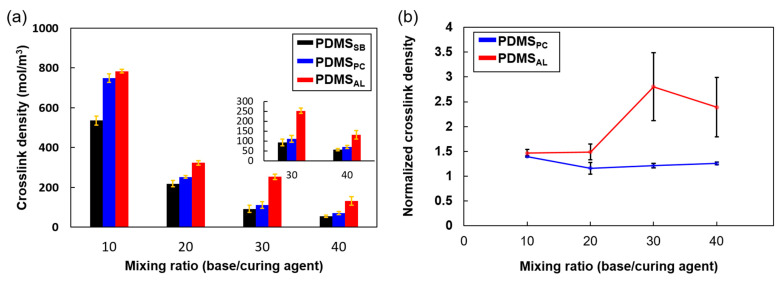
(**a**) Crosslink density estimated using the Flory–Rehner equation for different mixing ratios of bulk PDMS used for adhesion with various bonding techniques. (**b**) The normalized crosslink density of PDMS_PC_ and PDMS_AL_, defined by dividing their crosslink density by that of PDMS_SB_.

To quantify the crosslink density, we employed the Flory–Rehner equation, which provides a quantitative relationship between the swelling behavior of a polymer network and its crosslink density. Figure 5a and Table 2 show the crosslink densities estimated using the method described in the Experimental Section. As expected, the bulk PDMS involved in adhesion exhibited higher crosslink densities with increasing crosslinker concentrations at mixing ratios of 40, 30, 20, and 10, regardless of the bonding method used. This demonstrates that the crosslink density was effectively controlled through various bonding techniques and increased in the order of PDMS_SB_ < PDMS_PC_ < PDMS_AL_, whereas it decreased as the mixing ratio increased.

When the crosslinker was introduced as an adhesive layer, the most significant changes in crosslink density occurred in the reverse order, except at a mixing ratio of 40, as shown in Figure 5b. Normalization was performed to accurately compare the changes in crosslink density across different samples using simply bonded specimens (PDMS_SB_) at each mixing ratio as a baseline. The relatively lower normalized crosslink density at a mixing ratio of 40 compared to that at a mixing ratio of 30 is likely due to the presence of an excess of free chains with high mobility, resulting in the incomplete formation of a fully crosslinked three-dimensional network within the bulk [20]. Despite sufficient crosslinking at the surface to promote interfacial crosslinking, the absence of a fully developed crosslinked network within the bulk, which occupies a larger volume, results in only a marginal increase in the overall crosslink density. Consequently, the network formed at the interface may lack robustness due to its insufficient connectivity with the bulk network.

### 3.3. Stiffening Behaviors Caused by Crosslinking at the Interface

**Figure 6:**
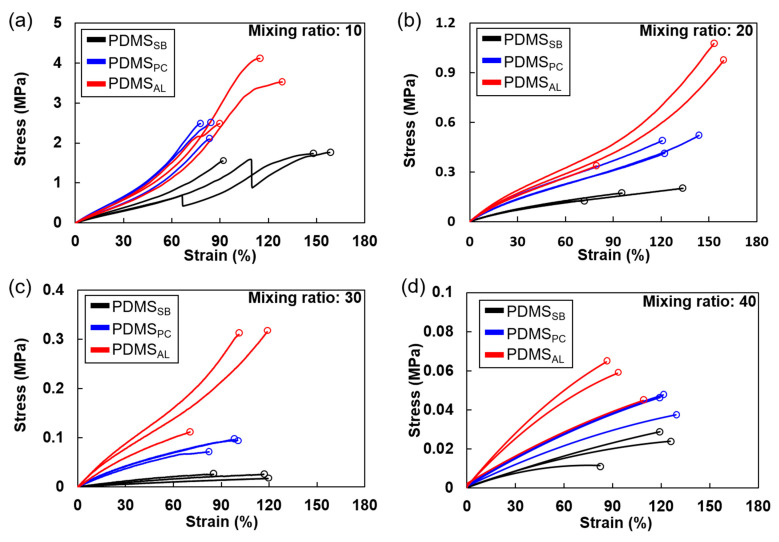
Stress–strain curves for various mixing ratios of bulk PDMS used in adhesion with different bonding techniques: (**a**) mixing ratio of 10, (**b**) mixing ratio of 20, (**c**) mixing ratio of 30, and (**d**) mixing ratio of 40. The stress drop observed in PDMS_SB_ in (**a**) indicates de-bonding occurred during the tensile test. Note the differences in scale among the four plots.

Variations in the interfacial crosslink density result in microstructural differences in PDMS, directly influencing its tensile behavior. Stiffening, a key characteristic feature of elastomers, is closely related to microstructural details, particularly the crosslink density [21]. A higher crosslink density generally increases the stiffness of the elastomer. As shown in Figure 6, the stiffening observed across the various bonding techniques demonstrates that the deposition of a crosslinker as an adhesive layer induces significant changes in mechanical behavior. Elastomers typically exhibit linear elasticity at low strains. However, as strain increases, the stiffening becomes more pronounced, particularly in systems with higher crosslink densities [ref]. For PDMS_AL_, significant stiffening was observed at strains exceeding 90% compared to PDMS_SB_ at mixing ratios of 20 and 30 (red lines vs. black lines in Figure 6b,c), which was not observed in PDMS_PC_ (blue lines in Figure 6b,c). From the crosslink density estimation in Section 3.2, it can be inferred that PDMS_AL_ exhibited a higher crosslink density than PDMS_SB_ and PDMS_PC_, resulting in a considerable increase in stiffness.

Moreover, when comparing the samples, PDMS_AL_ with a mixing ratio of 30 (red line in Figure 6c) exhibited stiffening even at a lower stress level across various strains than PDMS_PC_ with a mixing ratio of 20 (blue line in Figure 6b). This behavior indicates that although the inherent crosslink density of PDMS is lower, more extensive crosslinking occurs at the interface due to the presence of more mobile chains, resulting in stiffening even at lower stress levels [20]. In contrast, at a mixing ratio of 10, stiffening was consistently observed across all the bonding techniques (Figure 6a). This is attributed to the inherently high crosslink density within the material, suggesting that changes in the chemical structure at the surface are minimal and have a negligible impact on the overall tensile behavior. However, at a mixing ratio of 40, no distinct stiffening was observed within the examined strain range, despite additional crosslinking at the interface. This lack of stiffening can be attributed to the effect of the loosely crosslinked bulk PDMS, where stiffening typically occurs only at higher strains and requires greater deformation to sufficiently engage the polymer chains. This phenomenon is reminiscent of the marginal increase in overall crosslink density observed in PDMS_AL_ at a mixing ratio of 40, as discussed in the previous section, suggesting that an excessively low crosslink density in the bulk minimizes the impact of enhanced interfacial crosslinking on tensile behavior.

These findings highlight the critical role of the crosslink density at the interface and within bulk PDMS in determining the overall mechanical behavior of the material, particularly for different strain levels.

### 3.4. Tensile Properties of PDMS Considering Bonding Techniques

**Figure 7:**
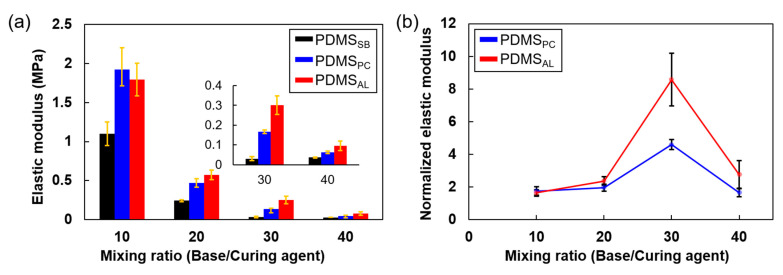
(**a**) Measured elastic modulus for different mixing ratios of bulk PDMS used for adhesion with various bonding techniques. (**b**) Normalized elastic modulus of PDMS_PC_ and PDMS_AL_, defined by dividing their elastic modulus by that of PDMS_SB_.

The effect of the crosslinker on interfacial adhesion was evaluated from a mechanical perspective. Figure 7a and Table 3 present the elastic moduli of the different bonding techniques. Since the elastic modulus is closely related to the crosslink density (i.e., an increase in crosslink density typically results in a higher elastic modulus due to the denser and more interconnected polymer network structure), the results align with those obtained from the crosslink density estimations.

Across all mixing ratios, PDMS_PC_ exhibited a higher elastic modulus than PDMS_SB_. This increase in elastic modulus can be attributed to the formation of additional crosslinks within bulk PDMS, which were not fully developed due to the short initial curing time.

For mixing ratios of 20, 30, and 40, when a crosslinker was used as an adhesive layer for bonding (PDMS_AL_), the elastic modulus was significantly enhanced compared to other bonding techniques, such as PDMS_SB_ and PDMS_PC_. This enhancement is likely due to the higher crosslink density of PDMS_AL_. However, at a mixing ratio of 10, this increase in elastic modulus was not observed. At this ratio, the high crosslinker concentration induced extensive crosslinking within the bulk PDMS (rather than at the interface), limiting further crosslinking potential at the interface due to the formation of shorter chains. Thus, the enhancement in material properties at a mixing ratio of 10 was primarily due to increased crosslink density within bulk PDMS rather than the effect of crosslinker deposition at the interface.

In Figure 7, the elastic modulus is normalized based on the elastic modulus of the simply bonded PDMS_SB_. At a mixing ratio of 30, the normalized elastic modulus showed the most significant improvement, likely due to an effective balance between chain mobility and the availability of crosslinking sites in bulk PDMS with a lower crosslink density. Specifically, as the mixing ratio increased from 10 to 30, the enhancement in elastic modulus became more pronounced, with an approximately 140% (2.4-fold) increase at a ratio of 20 and a 760% (8.6-fold) increase at a ratio of 30, compared to a mixing ratio of 10. Within this range, higher mixing ratios contributed to increased crosslinker deposition on the surface. However, at a mixing ratio of 40, the normalized elastic modulus decreased to approximately 160% (2.6 times) of the initial value. This reverse trend is due to the excessively low concentration of crosslinkers in bulk PDMS, which leads to a poorly formed crosslinked network. Consequently, the free chains engage in chain interdiffusion and crosslinking at the interface. Despite the significantly increased crosslink density and formation of a crosslinked network at the interface, these structures remained isolated from the bulk PDMS network. This reduction in the normalized elastic modulus is likely to continue as the mixing ratio exceeds 40. These results highlight the delicate balance required between bulk and interfacial crosslink densities to achieve effective tensile performance, underscoring the potential of controlled crosslinking strategies to enhance the mechanical properties of PDMS. Overall, our experimental observations are consistent with the mechanisms proposed in Section 3.1. The tensile properties of PDMS can be effectively improved by adjusting the crosslink density.

## 4. Conclusions

In this study, we investigated the influence of bonding techniques on the tensile properties of polydimethylsiloxane (PDMS), emphasizing the role of interfacial crosslink density in enhancing mechanical performance. By employing three distinct bonding methods—simply bonded (PDMS_SB_), post-cured (PDMS_PC_), and crosslinker-assisted bonding (PDMS_AL_)—we demonstrated that the incorporation of crosslinkers as an intermediate adhesive layer significantly enhances tensile properties by forming a robust three-dimensional crosslinked network at the PDMS—PDMS interface.

Our findings reveal that a crosslinker mixing ratio of 30 achieves the best mechanical performance, with the elastic modulus increasing by up to 760% compared to simply bonded PDMS. This enhancement is attributed to a precise balance between chain mobility and the availability of crosslinking sites, which promotes effective chain interdiffusion and covalent bonding at the interface. Conversely, at higher crosslinker concentrations (specifically, a mixing ratio <30), excessive crosslinking within the bulk PDMS restricts chain mobility, diminishing the efficacy of interfacial bonding and reducing the overall mechanical improvement.

These results underscore the necessity of balancing bulk and interfacial crosslink densities to achieve the desired material properties in PDMS systems. By systematically refining the parameters, we developed a scalable and versatile bonding strategy that significantly enhances tensile properties, offering transformative potential for applications requiring tailored mechanical behaviors, such as flexible electronics, microfluidics, and biomedical devices.

However, the use of other crosslinking compounds, such as vinyl-terminated oligomers, fillers, or catalysts, should also be considered to broaden the applicability and fine-tune the mechanical and interfacial properties of PDMS. Additionally, understanding the long-term stability of these enhanced interfaces under dynamic or cyclic loading conditions is essential for ensuring durability in practical applications. This includes addressing the effects of environmental and processing conditions on the interfacial bonding mechanism.

In summary, this study provides a robust framework for tailoring the tensile behavior of PDMS by strategically adjusting the interfacial crosslink density. The proposed bonding technique enables the precise tuning of localized mechanical properties, paving the way for the development of substrates with composite-like behavior tailored to meet the demands of advanced multifunctional applications in a wide range of fields [22].

## Figures and Tables

**Table 1:** Swelling ratios at equilibrium according to PDMS mixing ratios and bonding techniques.

Weight Swelling Ratio (%) at Equilibrium			
Mixing Ratio	PDMS_SB_	PDMS_PC_	PDMS_AL_
10	78.36 ± 2.61	59.13 ± 1.49	56.82 ± 0.55
20	153.72 ± 7.71	138.44 ± 3.01	115.6 ± 2.94
30	276.9 ± 34.42	243.8 ± 24.57	138.48 ± 5.52
40	383.05 ± 25.17	329.13 ± 23.87	218.73 ± 23.81

**Table 2:** Estimated crosslink density according to PDMS mixing ratios and bonding techniques.

Crosslink Density (mol/m^3^) at Equilibrium			
Mixing Ratio	PDMS_SB_	PDMS_PC_	PDMS_AL_
10	536.12 ± 22.07	749.37 ± 21.55	784 ± 8.59
20	218.59 ± 15.58	252.92 ± 7.67	324.04 ± 11.14
30	92.5 ± 17.58	111.73 ± 17.01	253.05 ± 14.07
40	55.74 ± 5.01	70.13 ± 7.92	131.72 ± 21.38

**Table 3:** Measured elastic modulus according to PDMS mixing ratio and bonding techniques.

Elastic Modulus (~30%) (MPa)			
Mixing Ratio	PMS_SB_	PDMS_PC_	PDMS_AL_
10	1.10 ± 0.15	1.92 ± 0.28	1.80 ± 0.21
20	0.24 ± 0.01	0.47 ± 0.05	0.57 ± 0.06
30	0.11 ± 0.02	0.41 ± 0.03	0.76 ± 0.14
40	0.03 ± 0.00	0.05 ± 0.01	0.09 ± 0.03

## Data Availability

The original contributions presented in the study are included in the article, further inquiries can be directed to the corresponding author.
